# Acute abdomen after vertebroplasty-A rare complication

**DOI:** 10.3389/fsurg.2022.1048107

**Published:** 2023-01-06

**Authors:** Xiao-ming Zhao, Xiao-xiao Lou, An-fa Chen, Yin-gang Zhang

**Affiliations:** Department of Orthopaedics of the First Affiliated Hospital, Medical School, Xi’an Jiaotong University, Xi’an, Shaanxi, China

**Keywords:** osteoporotic vertebral compression fractures, vertebroplasty, bone cement leakage, pancreatitis, Case report, acute abdomen

## Abstract

**Introduction:**

In recent years, as the concept of minimally invasive treatment has been accepted by the majority of patients, the application of percutaneous vertebroplasty in osteoporotic vertebral compression fractures has gradually increased, and research on the adverse complications of bone cement leakage has gradually deepened.

**Case:**

Here, we report a rare case of acute pancreatitis after vertebroplasty. The patient had no previous history of pancreatitis and presented with obvious abdominal pain after vertebroplasty. Abdominal CT examination revealed that the leaking bone cement penetrated the anterior wall of the L1 vertebral body into the diaphragm, and the heat released by the polymerization reaction caused inflammation and damage to the adjacent pancreas, resulting in poor blood flow to the pancreatic tissue and leading to acute pancreatitis. Early postoperative symptomatic treatment was given to the patient, and the corresponding symptoms were gradually relieved. During postoperative follow-up, the leaking cement did not degrade, but the patient had no symptoms.

**Conclusion:**

Lesions of adjacent organs caused by bone cement leakage are rare, and clinicians often ignore the association between such complications and vertebroplasty. This case report will provide guidance and a reference for clinicians.

## Introduction

Vertebroplasty is a minimally invasive surgical method for the treatment of osteoporotic vertebral compression fractures. With the advantages of simple operation, high safety, accurate analgesic effect, rapid pain relief, early postoperative activities, and the avoidance of various bed complications, it is widely used in clinical practice by an increasing number of spine surgeons (1). However, vertebroplasty requires the injection of bone cement into the affected vertebra through percutaneous puncture, so complications associated with cement leakage are also frequent. Most bone cement leaks are asymptomatic (2). In the past, several studies have reported serious complications of pulmonary embolism caused by bone cement leakage to the inferior vena cava (3). However, it is rare that bone cement leakage causes lesions in adjacent organs, and inexperienced doctors may overlook the association between these conditions and vertebroplasty. Recently, a case of acute pancreatitis caused by intraoperative bone cement leakage occurred in our department. The detailed medical records are reported as follows.

## Case presentation

**Figure 1:**
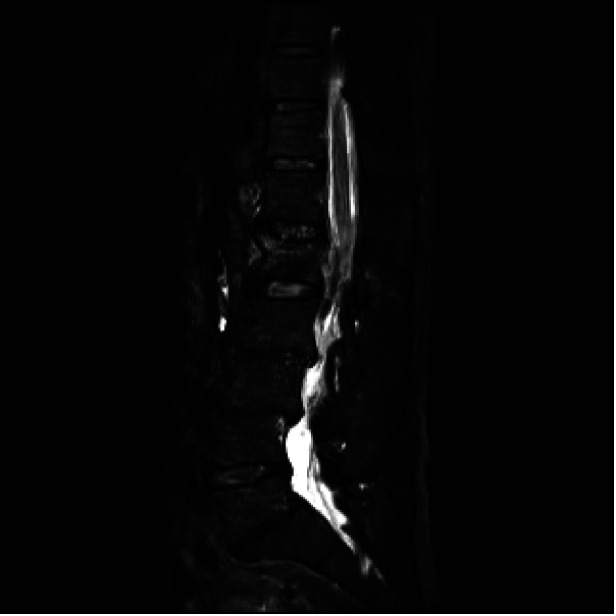
MRI showing fresh compression fracture of L1 vertebrae.

**Figure 2:**
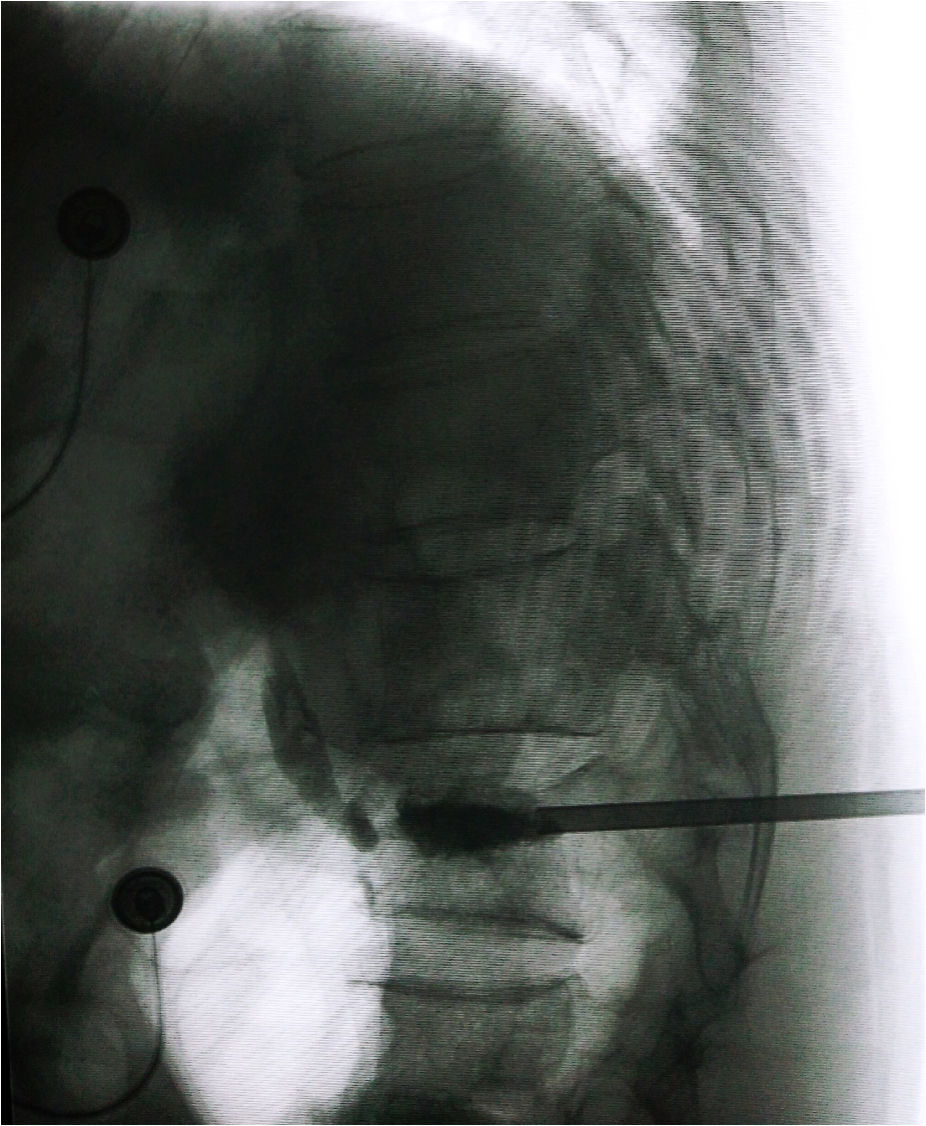
Intraoperative C-arm fluoroscopy showed bone cement penetrating the anterior wall and leaking into the surrounding area.

**Figure 3:**
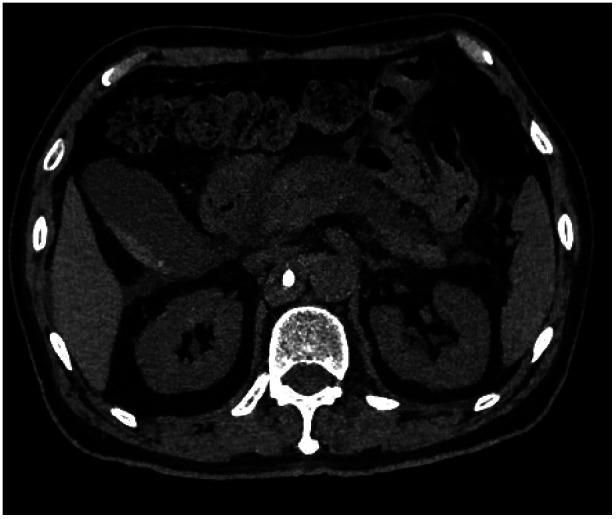
Abdominal CT showing obvious peripancreatic effusion with fuzzy edges, and high density bone cement shadow was seen in the right anterior of L1 vertebra.

**Figure 4:**
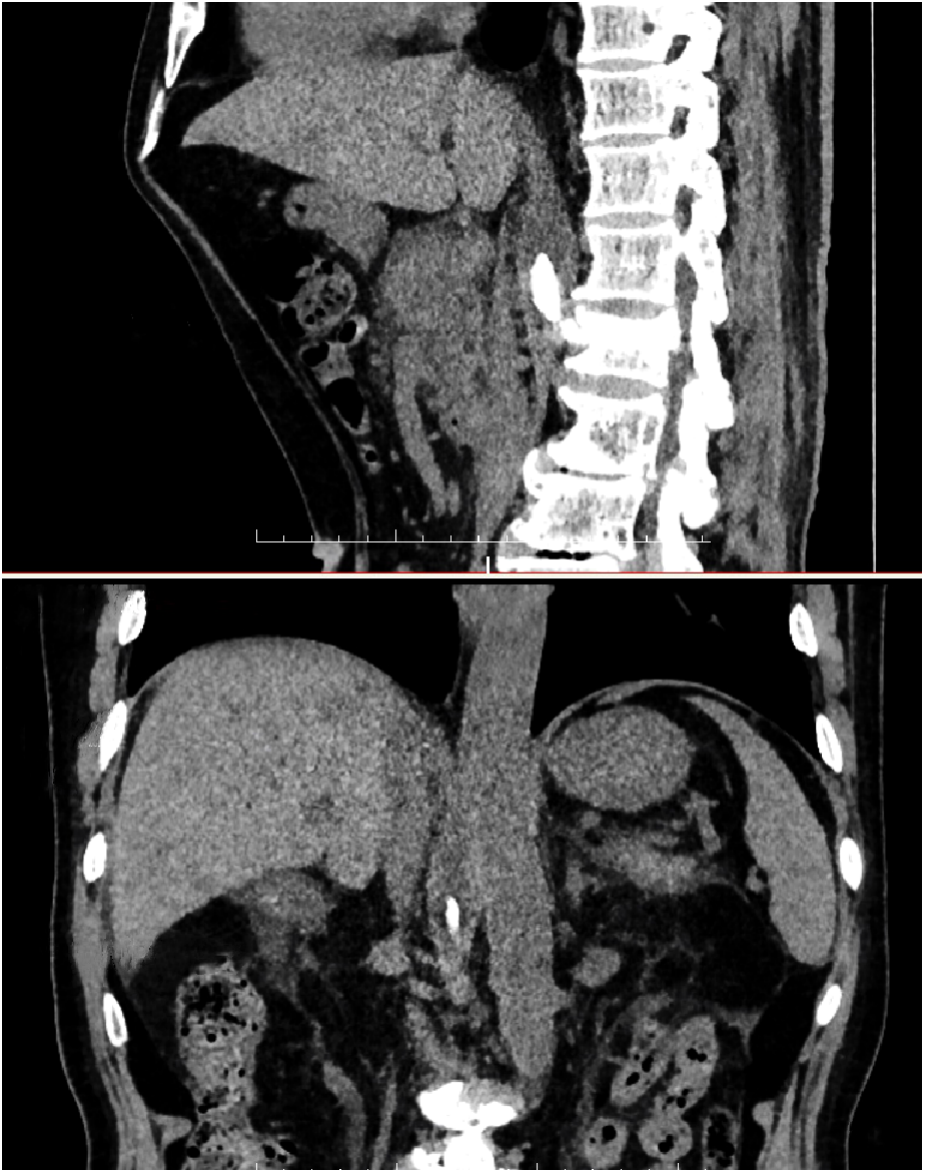
Postoperative 3D reconstruction showing that the cement was located in the right anterior of L1 vertebra, adjacent to the pancreas.

**Figure 5:**
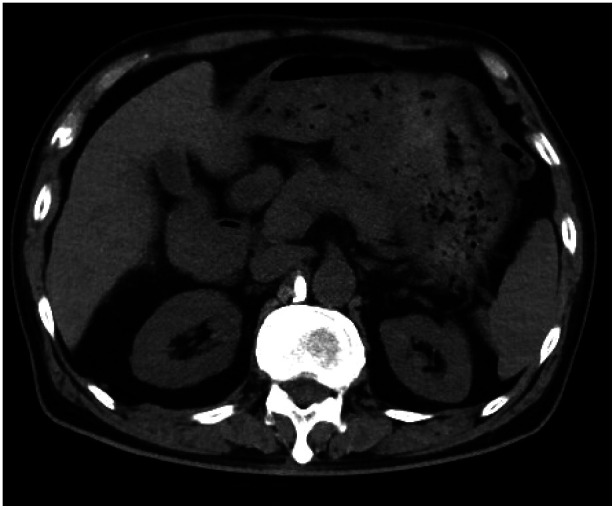
Abdominal CT showed no signs of pancreatitis and bone cement remained inside the diaphragm at 6 months of follow-up.

A 65-year-old man complained of chest and back pain, with mild pain when standing and walking and obvious pain when turning over in bed. After admission, the physical examination showed that there were no abnormalities in the heart, lung or abdomen, mild kyphosis of the lumbar spine, obvious tenderness at the spinous process of the L1 vertebral body, and no neurological radiation symptoms of the lower limbs. Preoperative MRI of the thoracic and lumbar vertebrae confirmed the diagnosis of fresh compression fracture of the L1 vertebrae (Figure 1). Therefore, he was scheduled for L1 vertebroplasty. During the operation, the diseased vertebral body was located by C-arm fluoroscopy, and 3 ml of bone cement was injected as usual. Then, we immediately stopped the injection. C-arm examination was performed again, and it was found that there was partial leakage of bone cement at the anterior edge of the vertebral body (Figure 2). We carefully observed the patient's vital signs and found no obvious symptoms. Therefore, we cleaned and bandaged the wound and returned to the ward for intensive monitoring. The day after surgery, the patient suddenly developed abdominal pain. Physical examination revealed left upper abdominal deep tenderness. Urgent examination of routine blood, electrolyte and amylase showed leukocyte 11*109/L and blood amylase 122.6 Abdominal Doppler ultrasonography revealed peripancreatic effusion. Abdominal CT showed obvious peripancreatic effusion, increased pancreatic volume with fuzzy edges, and a high-density bone cement shadow in the right anterior L1 vertebra (Figure 3). Three-dimensional reconstruction showed that the cement was located in the diaphragm, thickened and adjacent to the inferior vena cava and abdominal aorta (Figure 4). Given that the patient had no abnormality in preoperative examination and no previous history of pancreatitis and that the pancreas was located behind the peritoneum and in front of lumbar vertebral body 1, we speculated that the thermal effect and corresponding inflammatory reaction of bone cement leaking through the anterior vertebral wall during surgery may directly damage pancreatic tissue and cause acute pancreatitis. The patient was immediately treated with fasting, gastrointestinal decompression, acid inhibition, fluid rehydration, etc. Four days later, the patient's condition improved significantly, and all indexes were normal. At 6 months of follow-up, abdominal CT showed that the pancreas was normal in shape, and the bone cement in the diaphragmatic crura still existed, but the thickening of the diaphragm was significantly reduced (Figure 5).

## Discussion

Percutaneous vertebroplasty is one of the main methods for the treatment of osteoporotic vertebral compression fractures. It is widely used in clinical practice because of its obvious efficacy in enhancing the strength of the affected vertebral body, restoring the height of the vertebral body, relieving pain and improving the quality of life of patients compared with conservative treatment. However, with the deepening of research, the resulting complications have also been increasingly studied by scholars, and bone cement leakage is one of the most common complications (4). According to the different locations of bone cement leakage, it can be divided into six types: pervertebral leakage, intraspinal leakage, intervertebral foraminal leakage, intervertebral space leakage, paraspinal soft tissue leakage and mixed leakage (5). Direct leakage of bone cement into the vascular system is the most serious complication of vertebroplasty, which may cause pulmonary embolism, cerebral infarction, right heart embolism or peripheral venous system organ disorders (6, 7). To the best of our knowledge, in the current cases of bone cement leakage, there are few reported cases causing lesions of adjacent organs and tissues, which are often ignored by clinicians because the symptoms are not serious and there is no reasonable explanation for the pathogenesis. In this case, a typical bone cement leaked into the paravertebral tissue during infusion, damaging the adjacent pancreas and resulting in signs of postoperative peripancreatic exudation.

Common reasons for bone cement leakage are improper puncture techniques and operating procedures. Repeated punctures or improper punctures resulting in rupture of the pedicle or posterior cortex may cause bone cement to leak out of the rupture into the spinal canal. In addition, some patients with fractures spread to the vertebral wall, resulting in an incomplete vertebral wall, which also increased the probability of bone cement leakage.

At the same time, premature needle removal after the injection or failure to insert the needle core after the injection easily causes cement leakage. Second, there is an inappropriate amount and timing of bone cement injection. The injection of bone cement should be performed in the late wire-drawing phase (8). Premature injection of bone cement can lead to surrounding leakage due to its fluidity. At the same time, there was a positive correlation between the amount of bone cement injection and the incidence of leakage. Experts suggested that the amount of bone cement injection in the thoracic vertebra and the amount of bone cement injection in the lumbar vertebra should be within 3 ml and 5 ml, respectively, to achieve satisfactory clinical results (9). Excessive bone cement injection would lead to a significant increase in the incidence of leakage.

In this case, the acute pancreatitis caused by bone cement leakage may be related to inaccurate intraoperative positioning, improper puncture operation and imperfect preoperative evaluation of the location of vertebral fracture. We analyzed the possible pathogenesis as follows: Due to the severe osteoporosis in the patient, during the injection of bone cement, the working channel or push rod easily breaks through the lateral wall of the pedicle and the vertebral body wall, resulting in leakage to the surrounding tissues. Due to the rapid solidification of the bone cement during the injection, the bone cement was confined to the diaphragm and did not lead to further diffusion and wider injury. However, because the pancreatic tissue is located in the anterior and upper part of the L1 vertebral body, the exothermic reaction of leaking bone cement monomer in the polymerization process may directly cause the injury and inflammatory reaction of pancreatic ducts and microvessels in the adjacent pancreatic tissue, which leads to poor blood flow and pancreatic edema and ultimately to the occurrence of acute pancreatitis.

This case has some defects. Before the operation, only x-ray and magnetic resonance examinations were performed to prove the existence of a fresh vertebral compression fracture, and there was no detailed evaluation of the CT examination. If there is bone destruction in the vertebral wall before operation, the leakage of bone cement during the operation has much to do with it, and more attention should be given to the timing of the operation. The amount of bone cement injection is not necessarily the key factor of leakage. For the follow-up clinical operators, the situation of vertebral fracture should be strictly evaluated before operation, and the timing of injection should be strictly grasped.

## Conclusion

At present, percutaneous vertebroplasty is a minimally invasive method for the treatment of osteoporotic vertebral fractures, and its efficacy has been verified by a large number of clinical trials. Although it is undoubtedly less traumatic and effective, the complications related to bone cement leakage caused by this operation still need to be carefully considered, and the incidence of related complications can be reduced through strict grasp of the operation points. However, for patients with atypical abdominal pain symptoms after surgery, clinicians should pay attention to the possibility of bone cement leakage injury to adjacent organs to avoid missed diagnosis and misdiagnosis resulting in the delay of the disease.

## Data Availability

The original contributions presented in the study are included in the article/Supplementary Material, further inquiries can be directed to the corresponding author/s.
