# Corrigendum: Acute abdomen after vertebroplasty-a rare complication

**DOI:** 10.3389/fsurg.2024.1509452

**Published:** 2024-11-15

**Authors:** Xiao-ming Zhao, Xiao-xiao Lou, An-fa Chen, Yin-gang Zhang

**Affiliations:** Department of Orthopaedics of the First Affiliated Hospital, Medical School, Xi'an Jiaotong University, Xi'an, Shaanxi, China

**Keywords:** osteoporotic vertebral compression fractures, vertebroplasty, bone cement leakage, pancreatitis, Case report, acute abdomen

A Corrigendum on Acute abdomen after vertebroplasty-a rare complication By Zhao X-m, Lou X-x, Chen A-f and Zhang Y-g. (2022) Front. Surg. 9. doi: 10.3389/fsurg.2022.1048107

## Incorrect funding

In the published article, there was an error in the Funding statement. [The funding: the Clinical Research Award of the First Affiliated Hospital of Xi’an Jiaotong University, China (No. XJTU1AF-CRF-2019-025), was completed before the article was published, so we should delete it and replace it with another fund]. The correct Funding statement appears below.

